# Label-free photoacoustic imaging of human palmar vessels: a structural morphological analysis

**DOI:** 10.1038/s41598-018-19161-z

**Published:** 2018-01-15

**Authors:** Y. Matsumoto, Y. Asao, A. Yoshikawa, H. Sekiguchi, M. Takada, M. Furu, S. Saito, M. Kataoka, H. Abe, T. Yagi, K. Togashi, M. Toi

**Affiliations:** 1 Department of Breast Surgery, Graduate School of Medicine, Kyoto University, 54 Shogoin-Kawaharacho Sakyo-ku, Kyoto, 606-8507 Japan; 2 Department of Diagnostic Imaging and Nuclear Medicine, Graduate School of Medicine, Kyoto University, 54 Shogoin-Kawaharacho Sakyo-ku, Kyoto, 606-8507 Japan; 3 Department of Orthopaedic Surgery, Graduate School of Medicine, Kyoto University, 54 Shogoin-Kawaharacho Sakyo-ku, Kyoto, 606-8507 Japan; 4 Department of Plastic and Reconstructive Surgery, Graduate School of Medicine, Kyoto University, 53 Shogoin-Kawaharacho Sakyo-ku, Kyoto, 606-8507 Japan; 5 Japan Science and Technology Agency, ImPACT Program, Cabinet Office, K’s Gobancho, 7, Gobancho, Chiyoda-ku, Tokyo, 102-0076 Japan; 6 Medical Imaging System Development Center, Canon Inc., 3-30-2 Shimomaruko, Ohta-ku, Tokyo, 146-8501 Japan

## Abstract

We analysed the vascular morphology of the palm using a photoacoustic tomography (PAT) instrument with a hemispherical detector array. The three-dimensional (3D) morphology of blood vessels was determined noninvasively. Overall, 12 females and 11 males were recruited as healthy volunteers. Their ages were distributed almost evenly from 22 to 59 years. In all cases, many vascular networks were observed just beneath the skin and were determined to be veins anatomically. To analyse the major arteries, the layer containing the subcutaneous venous network was removed from the image. The analysis focused on the common and proper palmar digital arteries. We used the curvature of these arteries as a parameter to analyse their morphologies. There was no significant difference in the curvature between genders when comparing the subjects as a whole. The blood vessel curvature increased with age. Good agreement was found between the 3D numerical analysis results and the subjective evaluation of the two-dimensional (2D) projection image. The PAT system enabled visualization of the 3D features of blood vessels in the palm and noninvasive analysis of arterial tortuousness.

## Introduction

Blood is conducted via blood vessels; arterial blood carries the necessary oxygen to each organ in the body, and venous blood is reoxygenated in the lungs. In addition to carrying oxygen, blood contains various components. Therefore, we can examine peripheral blood to identify the presence of visceral diseases and obtain an estimation of an individual’s lifestyle. The vessel structure itself possibly reflects how blood has flowed through its lumen for many years, except in congenital cases. For example, arteriosclerosis due to hypertension, diabetes, and hyperlipidaemia is caused by the mechanical effects of long-term shear stress on the arterial wall, oxidation by free radicals, and invasion of cells into the wall of blood vessels in response to inflammation^1^. Varicose veins in the lower limbs are caused by the dysfunction of venous valves caused by standing over long periods^2^. Osler nodules inform us of the possible presence of infectious endocarditis^3^, and Raynaud’s phenomenon indicates the malfunction of peripheral vessels^4^. From this information, it is possible to speculate that peripheral blood vessel morphology reflects the whole body condition. To understand the abnormal blood vessels associated with the above-mentioned acquired diseases, it is necessary to construct a database of healthy subjects with normal blood vessels and compare abnormalities with normal characteristics of people in the same age range. Although the two-dimensional X-ray angiography results of the abdominal aorta of patients with disease have shown that the curvature changes in individuals over 40^5^, it has not been confirmed by clinical research with comparisons to the morphology of the peripheral blood vessels of healthy subjects. This lack of confirmation is because conventional blood vessel imaging methods require invasive procedures, such as the use of angiographic contrast agents and/or radiation exposure. Noninvasive vascular imaging modalities, such as noncontrast magnetic resonance imaging and ultrasonic Doppler methods, do exist; however, their resolution is limited. Although the resolution of ultrasonic Doppler is reported to have been improved by the superb microvascular imaging method^6^, this improvement is limited to two-dimensional (2D) cross-sectional imaging in B mode. The capability of visualizing the precise three-dimensional (3D) morphology of blood vessels by pseudo-3D imaging scanning with linear ultrasound probes has limitations.

We have developed a photoacoustic (PA) imaging system to acquire images for the diagnosis of breast cancer^7–11^ and have demonstrated that 3D imaging can be realized with a resolution of less than 0.5 mm using the third prototype (PAI-03)^10,11^. We considered this device to be useful for detailed analyses for breast cancer and peripheral blood vessels, such as those in the palm. In predicting diseases by the morphology of blood vessels in peripheral tissues, it is necessary to consider the performance limits of imaging and search for appropriate analytical methods. In addition, to properly compare healthy people and diseased individuals, it is necessary to understand how females and males differ and how morphology changes due to ageing in healthy subjects. The blood vessels of healthy subjects are believed to be continuous without abnormal bending or disruptions caused by disease. In this paper, we report an analytical test of blood vessel structure for exploratory clinical research, focusing on the twisting and winding of blood vessels in healthy subjects to determine the shape of blood vessels in healthy subjects.

## Results

**Figure 1:**
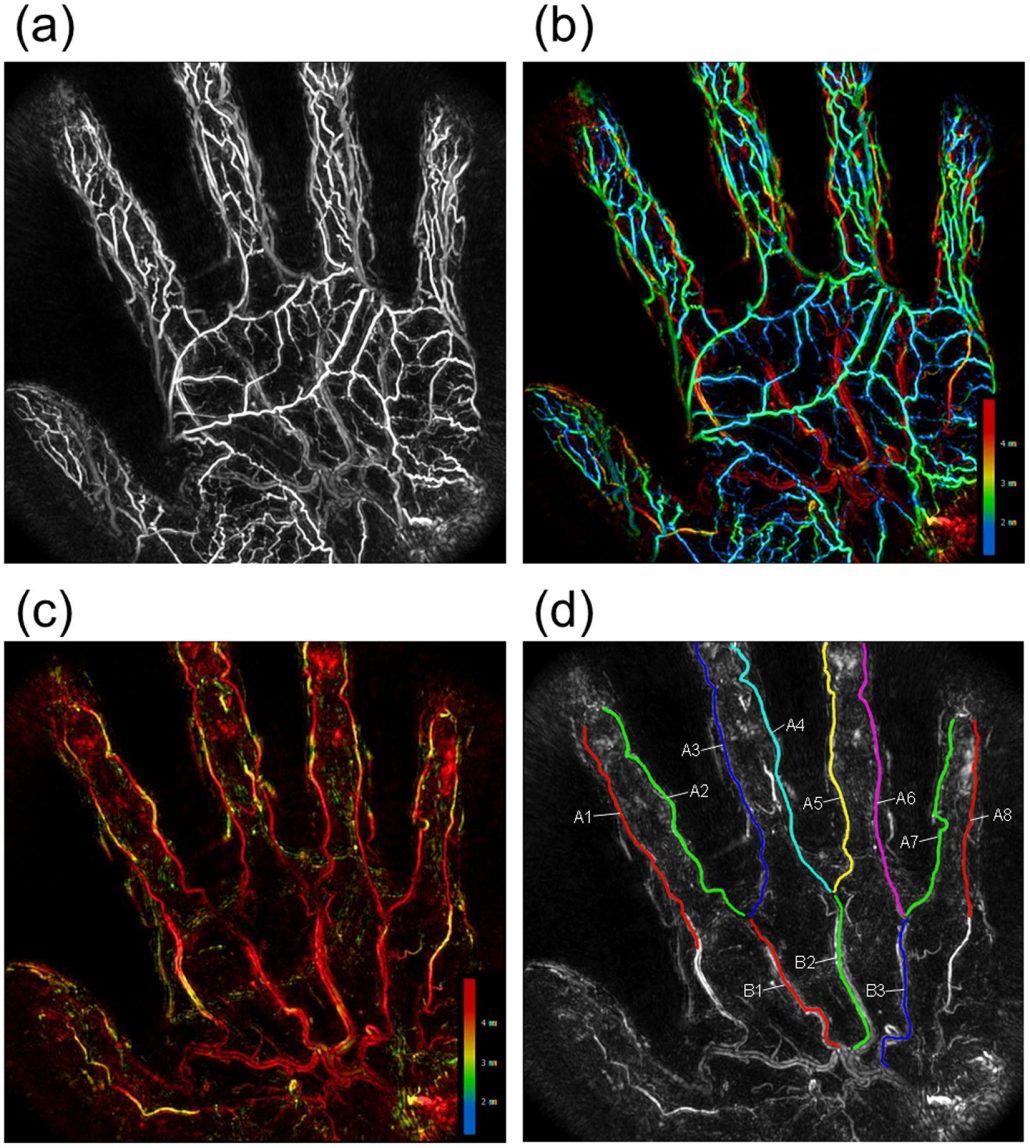
Example of a blood vessel image of the palm taken at a wavelength of 795 nm using the third prototype of our photoacoustic imaging system. (**a**) Complete maximum intensity projection (MIP) image of the hand; (**b**) image representing the depth as a colour parameter; (**c**) MIP image after deletion of the vein image near the skin surface; (**d**) superimposed image of the binarized coloured lines extracted from the common and proper palmar digital arteries over the original PA image. The original PA image as the background is a greyscale  image after deletion of the depth colour information from Fig. 1c. We labelled the three common palmar digital arteries as B1, B2, and B3 in order of arrangement starting from the radial side and labelled the eight proper palmar digital arteries as A1 to A8 in order of arrangement starting from the radial side of the index finger (see Supplementary movie 1).

Twenty-three healthy volunteers (12 females and 11 males) were recruited. We included several males and females in each 10-year age group between 20 and 60 years of age. The average age of the subjects was 37.9 years (22–56 years) for males and 38.5 years (25–59 years) for females. The subjects also included smokers and drinkers, but we did not analyse the differences in these lifestyle habits. PA images of the palms of the subjects were taken using PAI-03. Figure 1a shows an example of the maximum intensity projection (MIP) image of a photoacoustic tomography (PAT) image of a palm. The measurement wavelength was 795 nm.

First, surgeons and radiologists observed the PAT images in detail with a 3D viewer and identified the main arteries on the basis of their anatomical findings. In all cases, it was confirmed that the venous network was imaged in the layer just beneath the skin and the arteries were imaged deeper. Figure 1b, which is coloured according to the depth, shows that the superficial palmar arch, the common palmar digital arteries, and the proper palmar digital arteries were observed deeper than the network of veins. In contrast, the deep palmar arterial arch and the palmar metacarpal arteries, which were supposed to be located deeper, were not observed in the PAT image.

Figure 1c is an image obtained by deleting the network of veins near the skin from the data of Fig. 1b to easily distinguish these arteries. Figure 1d is an image obtained by superimposing the binarized coloured lines extracted from the common and proper palmar digital arteries over the original PA image. The original PA image as the background is a black and white image after deletion of the depth colour information from Fig. 1c. We labelled the three common palmar digital arteries as B1, B2, and B3 in order of arrangement starting from the radial side and labelled the eight proper palmar digital arteries as A1 to A8 in order of arrangement starting from the radial side of the index finger. This study focused on the common and proper palmar digital arteries because there are many individual differences in the shape of the superficial palmar arch^12^. The bendability of blood vessels was evaluated using the mean curvature of a complete finger artery.

**Figure 2:**
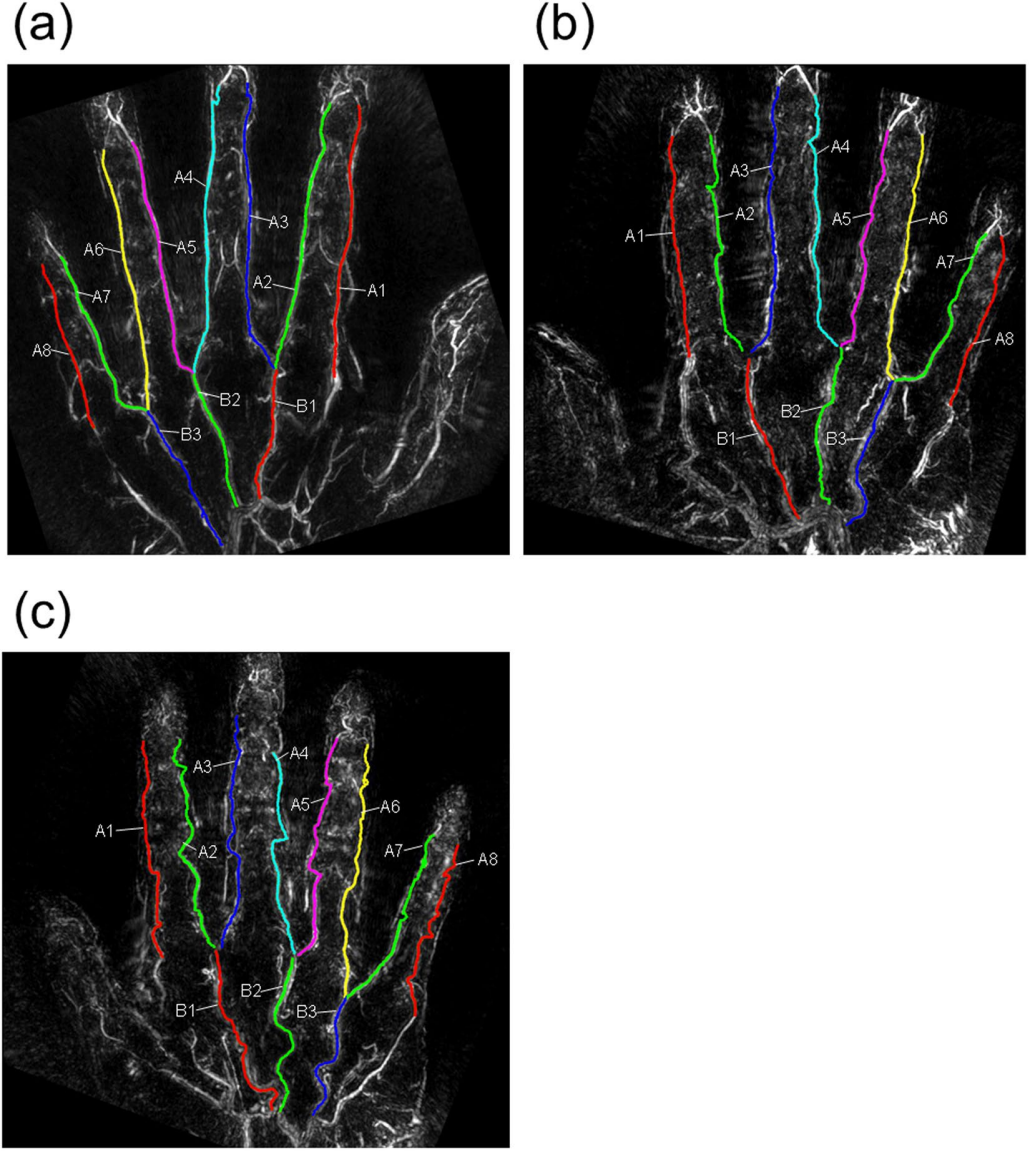
Typical examples of blood vessels with different curvatures. Three cases with different curvatures of the common and proper palmar digital arteries are shown. As shown in Fig. 1d, binary images of the common and proper palmar digital arteries are superimposed on the original PA image. (**a**) Example of a blood vessel with a small bend; (**b**) example of a blood vessel with a medium bend; (**c**) example of a blood vessel with a large bend.

**Figure 3:**
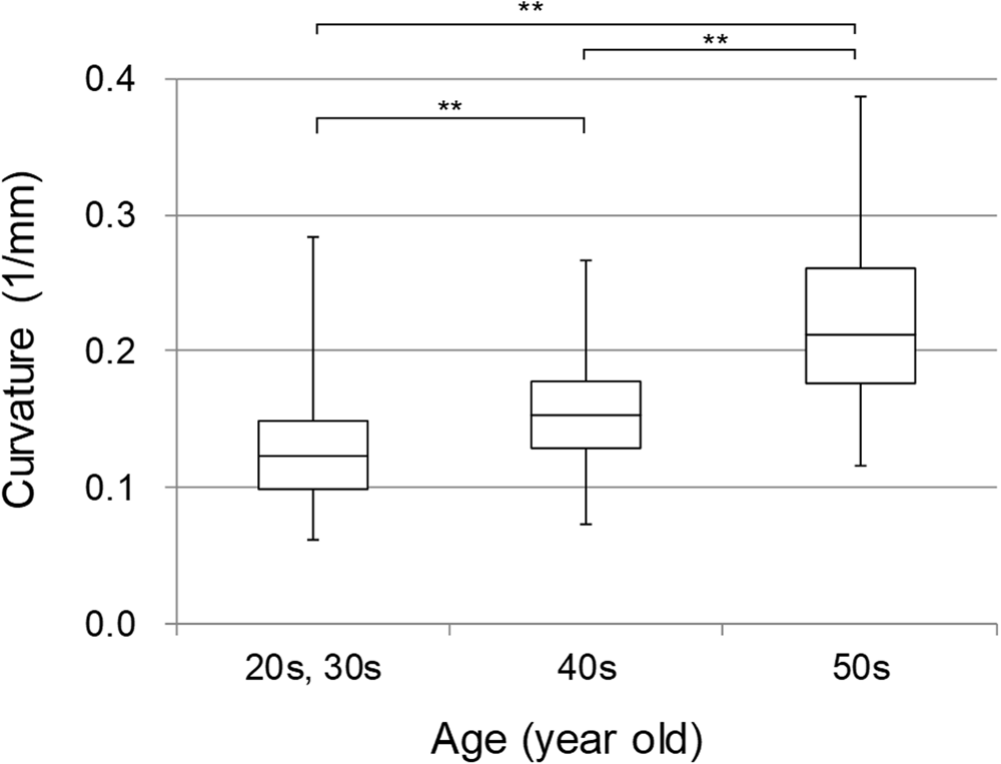
Results of statistical analysis of the curvature of the palmar arteries as a function of each age rage. Comparison was made in groups of individuals in their 30 s and under and those in their 40 s and 50 s. (**p < 0.01).

Figure 2a–c shows three cases with different curvatures of the common and proper palmar digital arteries. As is clear from these examples, the curvature of the blood vessels is different for each subject. We extracted blood vessels from the 3D data to obtain their average curvature. In this study, the eight proper palmar digital arteries and the three common palmar digital arteries were analysed as independent blood vessel data. The results for each age group are shown in Fig. 3. Here, we analysed subjects in their 20 s and 30 s as one group. We analysed the data without distinguishing between men and women in this figure. According to the results in Fig. 3, the curvature monotonically increased with age, and the differences between the groups were all significant. For the comparison without distinguishing between ages, there were no significant differences between genders or between the common and proper palmar digital arteries (Fig. S3a,b).

**Figure 4:**
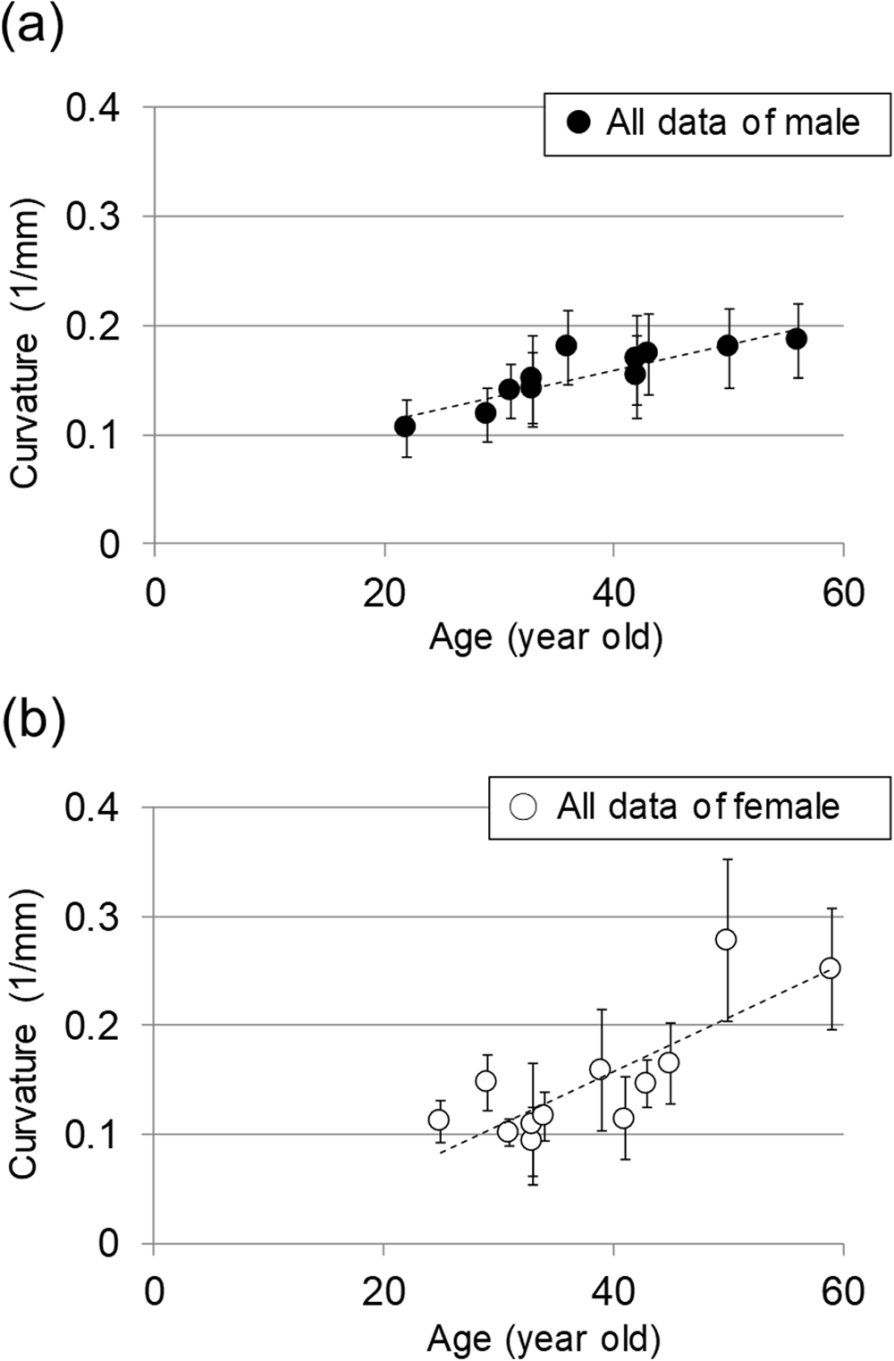
Results showing all curvature data as a function of age. (**a**) Results for male subjects. (**b**) Results for female subjects.

Figure 4a and b show the results of plotting all the data by gender. We can see that the curvature increases with age regardless of gender. Regarding the correlation between age and average curvature value, the Pearson correlation coefficient was 0.817 for males and 0.821 for females, both of which were significant (*p* < 0.01). For women, the curvatures of two individuals over 50 seemed to be large. When calculating the correlation with respect to the data of females except for those in their 50 s, the Pearson correlation coefficient was 0.563, and no statistically significant age correlation was obtained (*p* = 0.057). In the case of males, excluding those in their 50 s, the result was almost the same as for the all-age analysis. The Pearson correlation coefficient was 0.857, which was statistically significant (*p* < 0.01).

In the analysis of blood vessels in males, significant correlations were found between age and curvature in the proper palmar digital arteries in all evaluated blood vessels except A7. However, there was no significant difference in the common palmar digital arteries (Figs S4a–k and S6a). For females, there were significant correlations between age and curvature in the proper palmar digital arteries in all the blood vessels. B2 showed a significant tendency in the common palmar digital artery, but no significant tendency was obtained for B1 and B3 (Figs S5a–k and S6b).

Subsequently, the correlation between the results for the curvature obtained in 3D and the result of subjective evaluation by the evaluators of the 2D projection images was examined. Regarding the degree of agreement of the subjective evaluation results among the 10 evaluators, using the *W* value of Kendall, a value of 0.76 (*p* < 0.01) was obtained. Notably, the evaluation results of these 10 individuals were almost in agreement. The curvature and the average value of 10 subjective evaluation results were correlated. The correlation coefficients were 0.97 (*p* < 0.01) in females, 0.80 (*p* < 0.01) in males for the proper palmar digital arteries (Fig. S7a), 0.79 (*p* < 0.01) in females and 0.59 (*p* = 0.057) in males for the common palmar digital arteries (Fig. S7b). A significant correlation was obtained in every case except for the common palmar digital arteries of males.

## Discussion

We acquired blood vessel images of the palm noninvasively using the PAT apparatus and analysed the main arteries located in the shallow portion of the palm. In principle, haemoglobin’s oxygen saturation can be calculated from the absorption coefficient data at each wavelength because our PAT apparatus can record PA images at two wavelengths. However, because the palm has a complex organizational structure, it was difficult to calculate the light fluence *in vivo* and convert the obtained initial acoustic pressure value to the value of the absorption coefficient. Therefore, we could not perform the conversion. In this paper, we did not use the oxygen saturation level to identify the arteries and veins despite the acquisition of data of two wavelengths. Instead, based on the surgeon’s knowledge, we identified the blood vessels by their morphology with respect to the anatomically named arterial and venous networks at the surface. In this research, the anatomical analysis was performed on images acquired with just one wavelength (795 nm). An appropriate method to convert to the absorption coefficient in the future will possibly contribute to more accurate arteriovenous identification using haemoglobin oxygen saturation values even for microvessels without requiring anatomical knowledge.

The venous network of the subcutaneous surface layer was also depicted with high contrast, and it varied according to the subject (data not shown). This finding suggests the validity of personal authentication using the veins^13^. Because of this complexity, it was difficult to identify corresponding blood vessels for comparing subjects; therefore, we decided to exclude these veins from the analysis in this study.

It was impossible to identify deep blood vessels, such as the deep palmar arterial arch, by detailed observation because of the limitations of deep imaging inherent to the PAT apparatus used in this study. The imageable depth of the palm was less than 10 mm, which was less than half of the values of 24 to 27 mm obtained by the previous breast study^11^. The main cause for this difference is that the propagation of PA signals is obstructed by the presence of bone. In addition, complex structures such as muscles and tendons may affect the propagation of light and acoustic signals.

Although the main artery in a shallow location could be identified, the visibility of the artery varied among subjects. Skin conditions, such as horny skin and/or fat thickness, may have influenced the visibility on PAT. It will be necessary to further improve the signal-to-noise (SN) ratio to obtain clear images and to reproducibly analyse the deeper blood vessels (e.g., the superficial palmar arch) by decreasing system noise and improving the sensitivity of the ultrasound detectors. Regarding the morphology of the superficial palmar arch, although some were unclear, diversity, such as having loop-like structures or not, could be observed. Qualitatively, this result was in agreement with reports of the results for cadaveric hands analysed by 3D arteriography^12^. Although the clinical significance of this individual difference is unknown, accumulating data may make it possible to obtain some useful information.

Analysable images could be acquired with good reproducibility for the common and proper palmar digital arteries. In some subjects, the blood vessel image was observed to be missing along part of its course. It is difficult to assume that blood flow itself was disrupted because this study included healthy volunteers as subjects. Discontinuous blood vessels that nonetheless lie at a visible depth may be caused by a PAT-specific phenomenon called the limited-view problem^14^. When there is a stretch that it runs at an angle almost perpendicular to the scanning surface of the probe due to the bending of the blood vessel, the signal becomes weak or disappears. It may be necessary to examine images obtained with a posture that suppresses blind spots caused by the tortuousness of blood vessels, for example, by optimizing the angle of the finger during the PAT scan according to the examined lesion and its purpose.

According to the previous report^5^, an analysis was performed on the abdominal aorta of a person with a disease; the curvature was significantly increased from the age of 40 or more, and the gender difference was not significant. Although the number of samples was small in this paper, the increase in curvature due to ageing was significant. Although it is not appropriate to discuss the statistically significant difference due to the small sample size, the curvature increased in women in their 50 s. It is suggested that age and menopause are involved in the increase in cardiovascular disease according to the Framingham study^15^ and that oestrogen and progesterone exert cardiovascular protective effects^16^. In this preliminary study, it was suggested that the curvature data of males depends on ageing. It might be interesting to investigate the relationship between circulating steroid hormone levels and arterial curvatures particularly in individuals over 50.

The male data suggest a correlation between age and curvature except for the fifth finger (A7 in Table S1). In females, particularly in premenopausal women, this correlation was apparent only for the fifth finger (A6 in Table S2). The reasons for this opposing tendency depending on gender have not been clarified. Further data should be added in the future. There was no significance difference between males and females in the comparison of the whole blood vessels shown in Fig. S3a. With respect to blood vessel-specific data, the analysis shown in Fig. S6a,b illustrates a slightly different tendency between data from males and females. The validity of the result is not clear at the present stage due to the small number of cases; therefore, data to elucidate the cause should be added carefully. We plan on conducting additional clinical studies in healthy subjects using a new device with improved performance, such as better resolution, to analyse dozens of sampled cases in the future. In addition, we are planning clinical studies targeting disease groups to obtain knowledge of disease-specific vascular structures, including vein morphology.

A strong correlation was suggested between the numerical analytical results obtained for the curvature from 3D volume data and the results of the subjective evaluation using 2D projection images, particularly in the proper palmar digital arteries. In this numerical analysis, the blood vessels were extracted by a semi-automatic extraction method, which traces the blood vessel after its position is verified manually, as mentioned in the Methods. Technological developments will allow vessels to be extracted fully automatically by improving the PA apparatus and the image reconstruction technology and extractability using machine learning. However, when vessel extraction for numerical analysis, e.g., of deep blood vessels with a poor SN ratio, does not work well, substitution by the physician’s subjective evaluation may be possible. A search for more accurate and simple quantification methods is required, considering the accuracy necessary for blood vessel extraction and the desired diagnosis time.

Accumulating knowledge of the tortuousness of such blood vessels may provide important information not only to the medical field but also to the fields of health care and beauty. In future research, we intend to accumulate knowledge of the tortuousness of arteries by increasing the number of subjects and also by analysing veins that were not analysed this time. We expect these studies to provide further useful information.

In summary, it was possible to analyse the features of blood vessels noninvasively by PAT measurements of palms in healthy subjects. In this *in vivo* exploratory clinical study, we analysed the curvature of arteries in the human palm and found an age effect. The research achievement of measuring 3D images noninvasively, conveniently, and with high definition will be the trigger for a large-scale clinical trial aimed at screening for lifestyle diseases in the future. We will continue to improve the equipment and analytical techniques, contributing to research leading to vascular health.

## Materials, Subjects, and Methods

### Device configuration

We used the PAI-03 system with a hemispherical detector array^10,11^ made by Canon Inc. (Tokyo, Japan) in collaboration with Optosonics Inc. (USA). A palm of the subject was placed in the breast-holding cup (see Supplementary Fig. S1). A PA image was acquired by irradiating the tissue using a laser with light at wavelengths of 755 nm and 795 nm. For analysis of the data obtained in this clinical research, a PA image acquired at 795 nm with equal absorption coefficients of oxyhaemoglobin and deoxyhaemoglobin was used to analyse the blood vessel structure (the image corresponding to the total haemoglobin distribution). Since haemoglobin oxygen saturation was not analysed, images taken at a wavelength of 755 nm were not used. The specifications for the ultrasonic detectors set in the hemispherical detector array were the same as in our previous report, and the centre frequency was 2 MHz. Universal back-projection (UBP) was used for the PA image reconstruction^17^. The voxel size was 0.125 mm.

### Vascular evaluation image

To analyse the arteries of the palm, the venous network just below the skin was removed from the image data. At this time, “cloth simulation” was used to specify the position of the skin surface^18^. The depth of the removed venous network varied from subject to subject, but it was present at a position around 5 mm from the calculated skin surface. The location of the artery was determined by interpretation by a surgeon and a radiologist. This blood vessel was traced and binarized semi-automatically by the following method. On the MIP image in the 3D viewer^19^, several points on the artery were manually input by clicking with a mouse, and the 3D coordinates were acquired. For each interval delimited by adjacent points, the voxels with the highest likelihood of being a blood vessel were searched from both endpoints at fixed distances. The likelihood was calculated from the brightness value of the voxel, the attractive force from the terminal point, and the deviation angle of the travelling direction. Tracing was considered successful when the search points overlapped in the middle of the section. Next, the obtained coordinates were interpolated and connected to form a vessel travel route. In cases of failure, we added a guide point, and after correcting the position of the point, we attempted the process again. Finally, the moving average with respect to the route coordinates was calculated, and fine fluctuations in the route were eliminated.

### Calculating the curvature

The curvatures of the common palmar digital arteries were calculated for the whole blood vessel range. The curvatures of the proper palmar digital arteries were calculated from the bifurcation of the common palmar digital artery up to the position where the first blood vessel’s bifurcation beyond the distal interphalangeal (DIP) joint was confirmed. When the branch could not be observed, the calculation region was set by reference to the branch position of the adjacent proper palmar digital artery. For the finger arteries not branching on the palmar side from the common palmar digital artery (A1, A8), the calculation region was set by referring to the branching position of the adjacent artery. When the blood vessel was unclear because of the influence of noise, body movement, etc., the curvature was calculated only within the area that was clearly imaged. When part of the blood vessel was unclear and interrupted, if the doctor judged that it was obviously the same continuous blood vessel, it was interpolated smoothly. The curvature was obtained using the coordinate values of three points on the blood vessel traced by the above method (Fig. S2). Based on the surgeon’s experience that the radius of curvature is not smaller than the vessel diameter and the diameter of the blood vessel along the side of the finger is approximately 1 mm, the curvature value was obtained by using three coordinate values in a range of less than 1 mm on the blood vessel^20^. To calculate the curvature of a blood vessel, first, a smoothing process of discretized voxels was performed. The moving average was used for the smoothing process, and the moving average was 5 voxels. Data after the moving average were linearly interpolated to obtain a continuous centre line of the blood vessel. Three points (a reference point and the points before and after the point) for calculating the curvature at intervals of 0.5 mm were calculated along the centre line of the blood vessel, and the local curvature radius of the blood vessel at the reference point was calculated. Calculation of the local radius of curvature was performed for the entire finger artery, and the curvature value of the finger artery was determined by finding the average of the entire artery of interest. The curvatures were obtained for each of the three common palmar digital arteries and eight proper palmar digital arteries and were treated as independent data.

### Subjects

Subjects were recruited so that the number of males and females was almost the same and the number was almost the same among age groups. An exploratory clinical study was conducted among 23 subjects. Regarding the female subjects, the women under 40 years were all premenopausal. There were two women in their 50 s, one of whom was postmenopausal. The other refused to answer the question about menopause status. Therefore, it was a limitation of this study that the menopause status of one woman was not known. For reference, the average menopausal age of Japanese women has been reported to be approximately 50 years^21^.

The present study was approved by the Ethics Committee of the Kyoto University Graduate School of Medicine (UMIN000018893) and written informed consent was obtained from all subjects. This study was conducted in accordance with the Declaration of Helsinki.

### Method of subjective evaluation

The blood vessel image was evaluated by a total of 10 individuals, including four physicians and six medical researchers. When assessing a blood vessel image, the evaluation was performed on a PC screen using a two-dimensional MIP image in which subcutaneous veins were deleted from the image. Three level evaluation criteria images were prepared by surgeons and radiologists (Supplementary Figs S8 and S9).

### Statistics

With respect to the presence or absence of differences according to age, multiple comparisons were conducted using a nonparametric test with the Steel-Dwass method. Welch’s t test was used for comparison between the two groups. The Pearson correlation coefficient was used to determine the correlation between age and curvature. To investigate the variability among subject evaluators, the *W* value of Kendall’s coefficient of concordance was used.

## Electronic supplementary material

Supplementary materials

Supplementary movie
