# Supplementary material for: Label-free photoacoustic imaging of human palmar vessels: a structural morphological analysis
Source: Sci Rep. 2018 Jan 15;8:786. doi: 10.1038/s41598-018-19161-z (PMC5768743; doi:10.1038/s41598-018-19161-z)

Label-free photoacoustic imaging of human palmar vessels: a structural morphological analysis

Authors:
Y. Matsumoto[1], Y. Asao[1,5], A. Yoshikawa[1], H. Sekiguchi[2], M. Takada[1], M. Furu[3], S. Saito[4], M. Kataoka[2], H. Abe[6], T. Yagi[5], K. Togashi[2] and M. Toi[1]

Contributed equally to this work: Yoshiaki Matsumoto, Yasufumi Asao

Affiliations:
[1]Department of Breast Surgery, Graduate School of Medicine, Kyoto University, 54 Shogoin-Kawaharacho Sakyo-ku, Kyoto 606-8507, Japan
[2]Department of Diagnostic Imaging and Nuclear Medicine, Graduate School of Medicine, Kyoto University, 54 Shogoin-Kawaharacho Sakyo-ku, Kyoto 606-8507, Japan
[3]Department of Orthopaedic Surgery, Graduate School of Medicine, Kyoto University, 54 Shogoin-Kawaharacho Sakyo-ku, Kyoto 606-8507, Japan
[4]Department of Plastic and Reconstructive Surgery, Graduate School of Medicine, Kyoto University, 53 Shogoin-Kawaharacho Sakyo-ku, Kyoto 606-8507, Japan
[5]Japan Science and Technology Agency, ImPACT Program, Cabinet Office, K's Gobancho, 7, Gobancho, Chiyoda-ku, Tokyo 102-0076, Japan
[6]Medical Imaging System Development Center, Canon Inc., 3-30-2 Shimomaruko, Ohta-ku, Tokyo 146-8501, Japan
*To whom correspondence should be addressed: yoshiaki@kuhp.kyoto-u.ac.jp

Supplementary materials

Supplementary figures:

Fig. S1

Experimental setup of PAI-03. (a) Photograph of PAI-03 [11]. During measurements of the breast, the patient lies prone on the bed and places one breast in the spherically shaped cup indicated by the yellow arrow. (b) Photograph of palm measurements performed using this equipment. (c) Photograph of a palm that was set in the measurement cup. (d) Photograph of a palm being scanned. The subject was covered with a black cloth for safety to avoid the unexpected laser illumination of the patient's eye.

Fig. S2

Schematic for the calculation of the curvature used in this paper. For the sake of simplicity, this figure is shown in a two-dimensional plane, but the actual calculation was carried out in three-dimensional space. (a) Schematic of the blood vessel. (b) Enlarged figure for determining the moving average. (c) Figure of the linear interpolation and the calculation of the curvature. The curvature value was obtained by using three coordinate values in a range of less than 1 mm on the blood vessel. First, the coordinates of each point on the blood vessel were obtained by calculating the moving average (indicated by the red circle) of five discretized voxels (indicated by the red dashed line and arrow) centred on each point, as shown in Fig. S2 (b). By linearly interpolating the coordinates of the moving average points (shown by the red circles in Fig. S2 (b)), a continuous centre line of the blood vessel was created (shown by the red solid line in Fig. S2 (c)). Furthermore, along the centre line of the blood vessel, three points (the reference point and the nearest neighbouring point on each side) for calculating the curvature at intervals of 0.5 mm were calculated (shown by the blue circles in Fig. S2 (c)). The local radius of curvature of the blood vessel at the reference point was determined from the coordinates of the three points. The area from the reference point to the curvature centre point is displayed with a blue solid line, and the areas from the nearest neighbouring point on each side to the centre of curvature are indicated by blue dashed lines. The local curvature value at the reference point was obtained by calculating the reciprocal of the radius of curvature. Calculations of the local curvature were made for the entire finger artery, and the average value of the entire finger artery was taken as the curvature value of the finger artery in this paper. Curvature was calculated for each of the three common palmar digital arteries and the eight proper palmar digital arteries.

Fig. S3

Comparison of the curvature of the palmar arteries (a) between males and females and (b) between the

proper and common palmar digital arteries. (NS means not significant)

Fig. S4

Relationship between age and curvature of the palmar arteries of each finger in males: (a) A1, (b) A2, (c) A3, (d) A4, (e) A5, (f) A6, (g) A7, (h) A8, (i) B1, (j) B2 and (k) B3.

Fig. S5

Relationship between age and curvature of the palmar arteries of each finger in females: (a) A1, (b) A2, (c) A3, (d) A4, (e) A5, (f) A6, (g) A7, (h) A8, (i) B1, (j) B2 and (k) B3.

Fig. S6

Comparison of the curvature of the palmar arteries and subjective evaluation results of the tortuousness of the arteries. (a) Proper palmar digital arteries, (b) common palmar digital arteries. Subjective evaluation of the tortuousness of arteries was performed by 10 evaluators according to the representative sample shown in Fig. S8 and S9.

Fig. S7

Results showing the correlation coefficient for curvature and age obtained for each artery. The eight proper palmar digital arteries are labelled as A1 to A8 and the three common palmar digital arteries as B1, B2, and B3. All age data were analysed. (a) Result for males. (b) Result for females. (* $p < 0.05$).

Fig. S8

Evaluation criteria for the subjective evaluation of the proper palmar digital arteries. As shown in Fig. 1c, the subcutaneous vein network was evaluated with the deleted blood vessel image. For evaluations, black and white images without colour depth information were used. (a) Example of score 1 (straight) (b) example of score 2 (slight bend) and (c) example of score 3 (bend).

Fig. S9

Evaluation criteria for the subjective evaluation of the common palmar digital arteries. For evaluations, black and white images without colour depth information were used, as shown in Fig. S7. (a) Example of score 1 (straight), (b), (c) examples of score 2 (slight bends, 2 or fewer), (d) example of score 3 (strong bends, 2 or fewer), and (e) example of score 3 (3 or more bends).

Supplementary movie:

S_movie_1.avi. Movie showing rotation of the MIP image, as shown in Fig. 1.

Fig. S1

(a)

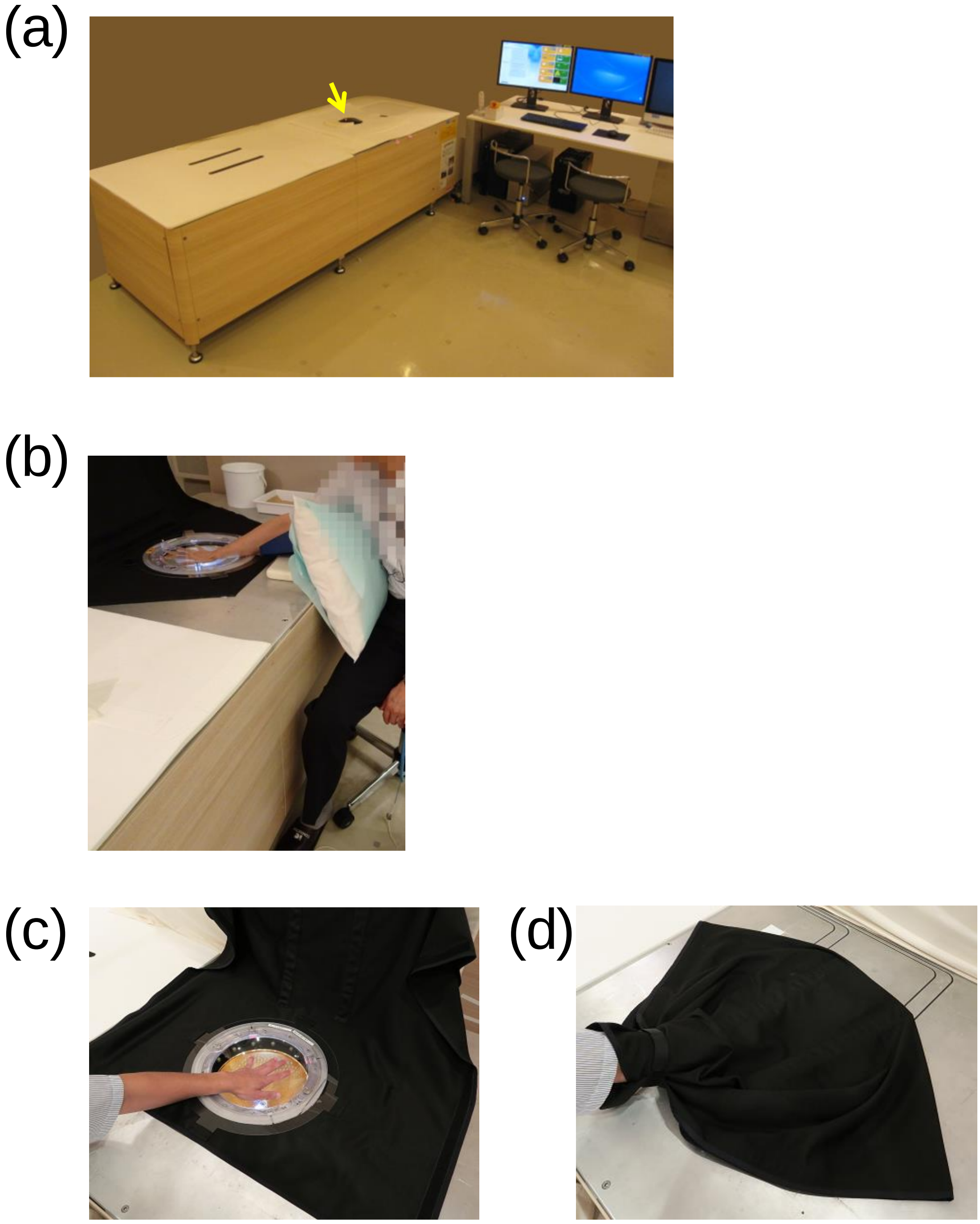

Fig.S2

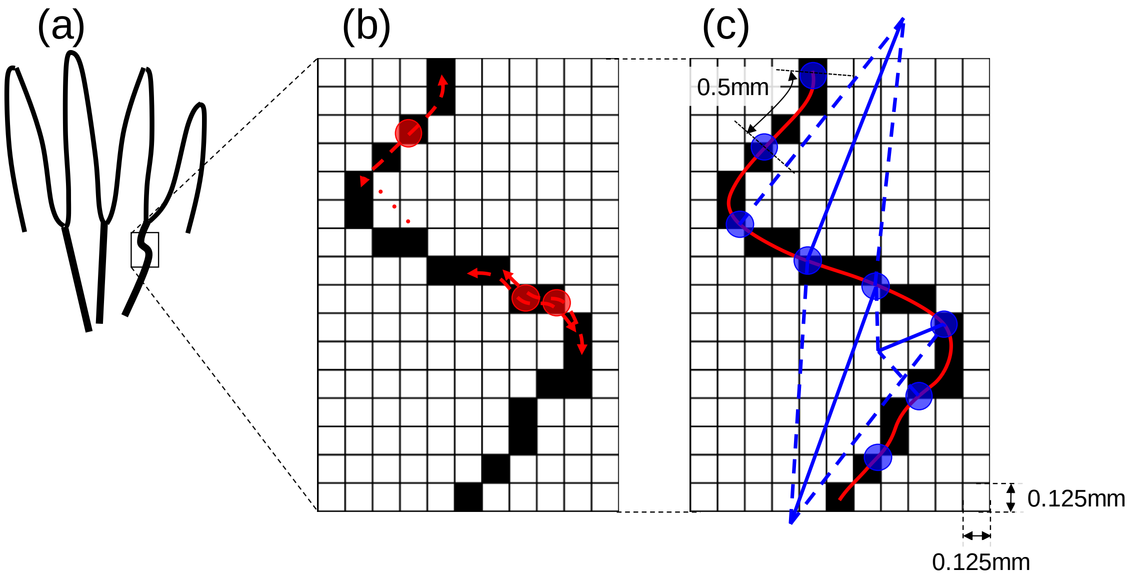

Fig. S3

(a)

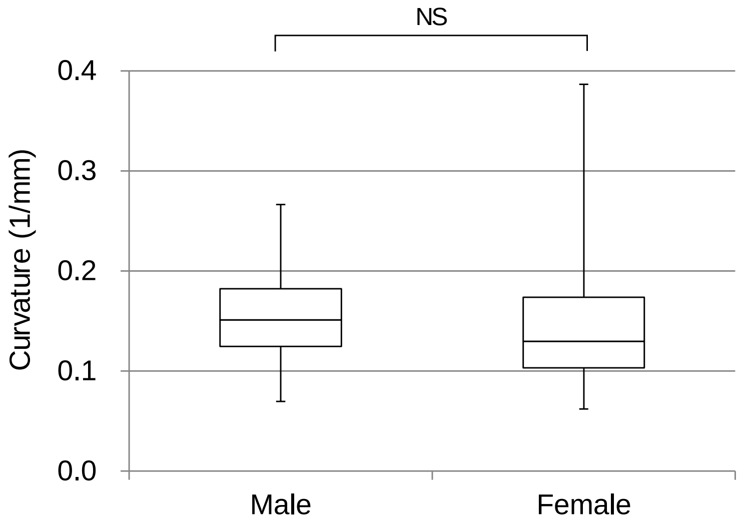
NS
0.4
0.3
0.2
0.1
0.0
Curvature (1/mm)
Male
Female

(b)

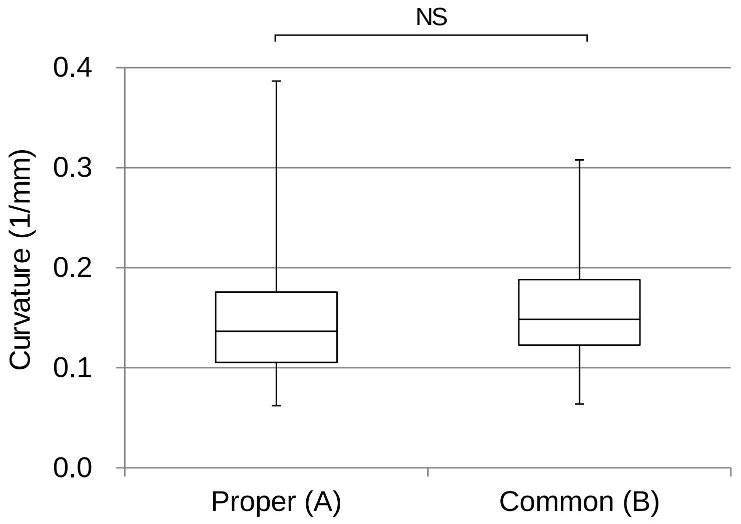
NS
0.4
0.3
0.2
0.1
0.0
Curvature (1/mm)
Proper (A)
Common (B)

Fig. S4

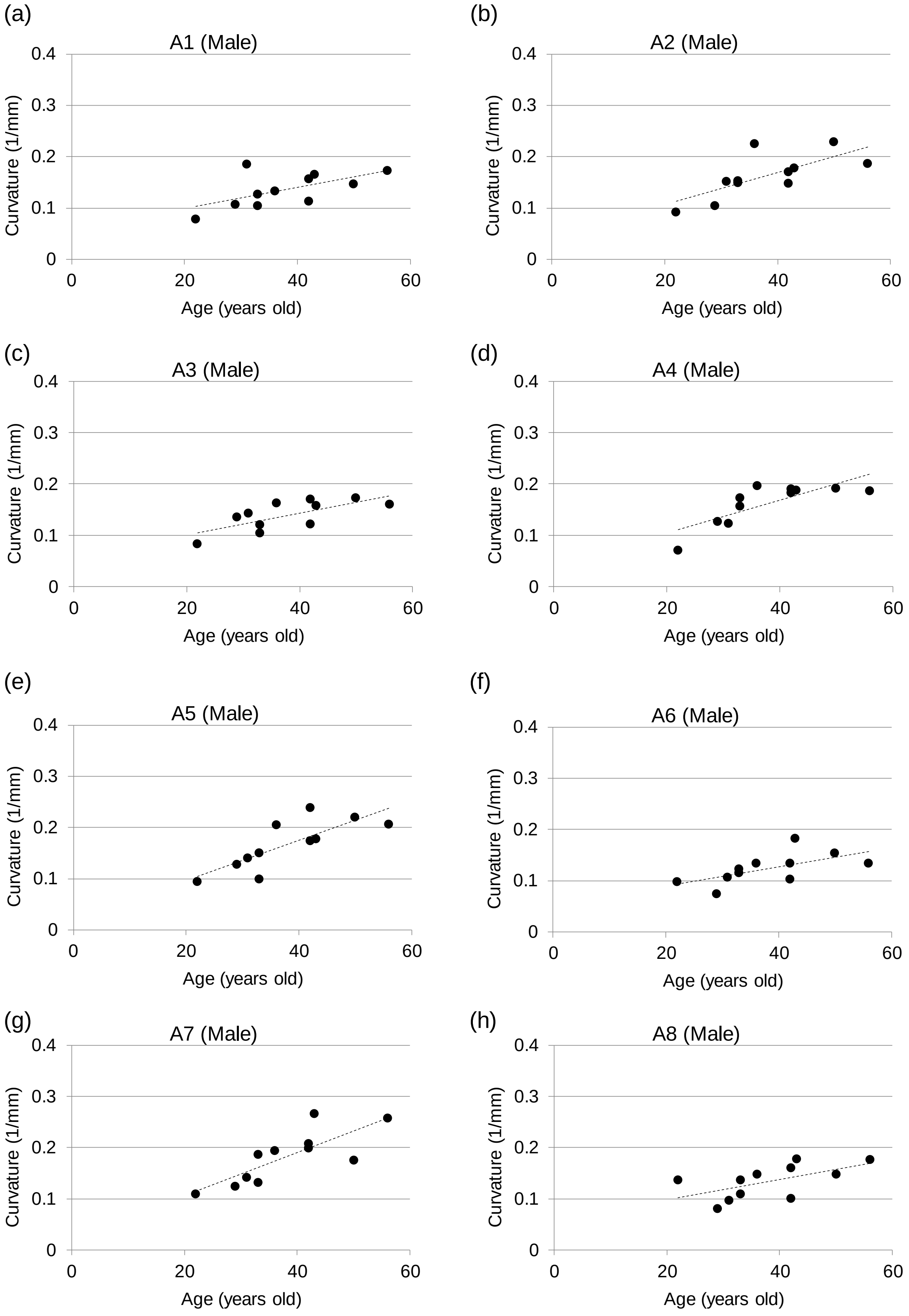
(a)
A1 (Male)
(b)
A2 (Male)
(c)
A3 (Male)
(d)
A4 (Male)
(e)
A5 (Male)
(f)
A6 (Male)
(g)
A7 (Male)
(h)
A8 (Male)
Curvature (1/mm)
0.4
0.3
0.2
0.1
0
Age (years old)
0
20
40
60

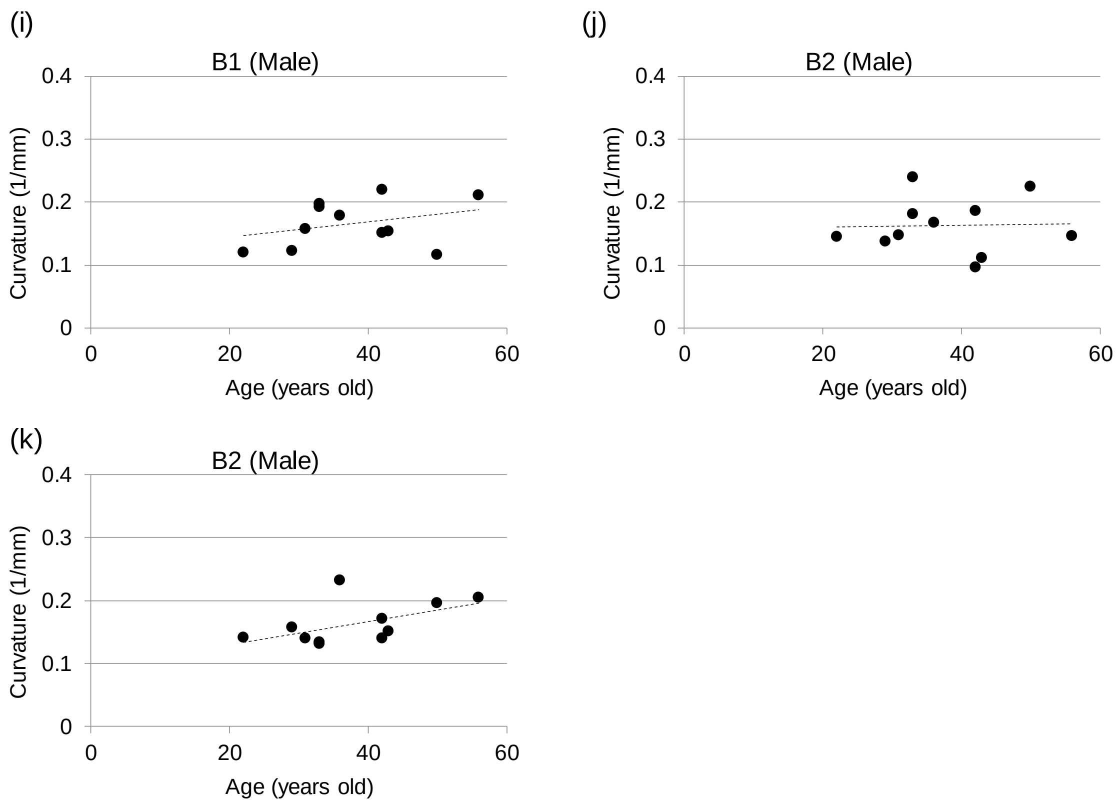
(i)
B1 (Male)
Curvature (1/mm)
Age (years old)
0.4
0.3
0.2
0.1
0
0
20
40
60
(j)
B2 (Male)
Curvature (1/mm)
Age (years old)
(k)
B2 (Male)
Curvature (1/mm)
Age (years old)

Fig. S5

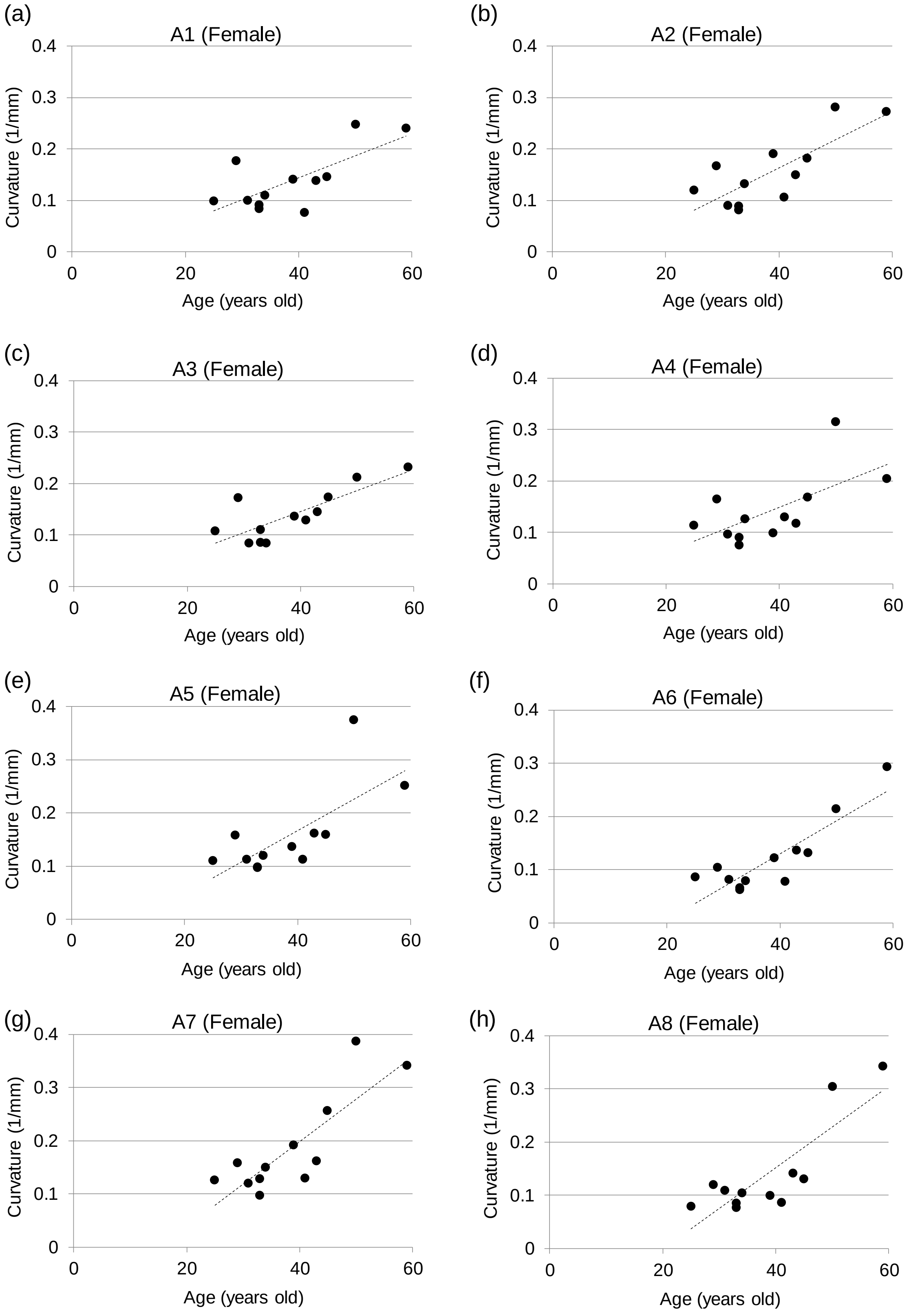
(a)
A1 (Female)
(b)
A2 (Female)
(c)
A3 (Female)
(d)
A4 (Female)
(e)
A5 (Female)
(f)
A6 (Female)
(g)
A7 (Female)
(h)
A8 (Female)
Curvature (1/mm)
Age (years old)
0
0.1
0.2
0.3
0.4
0
20
40
60

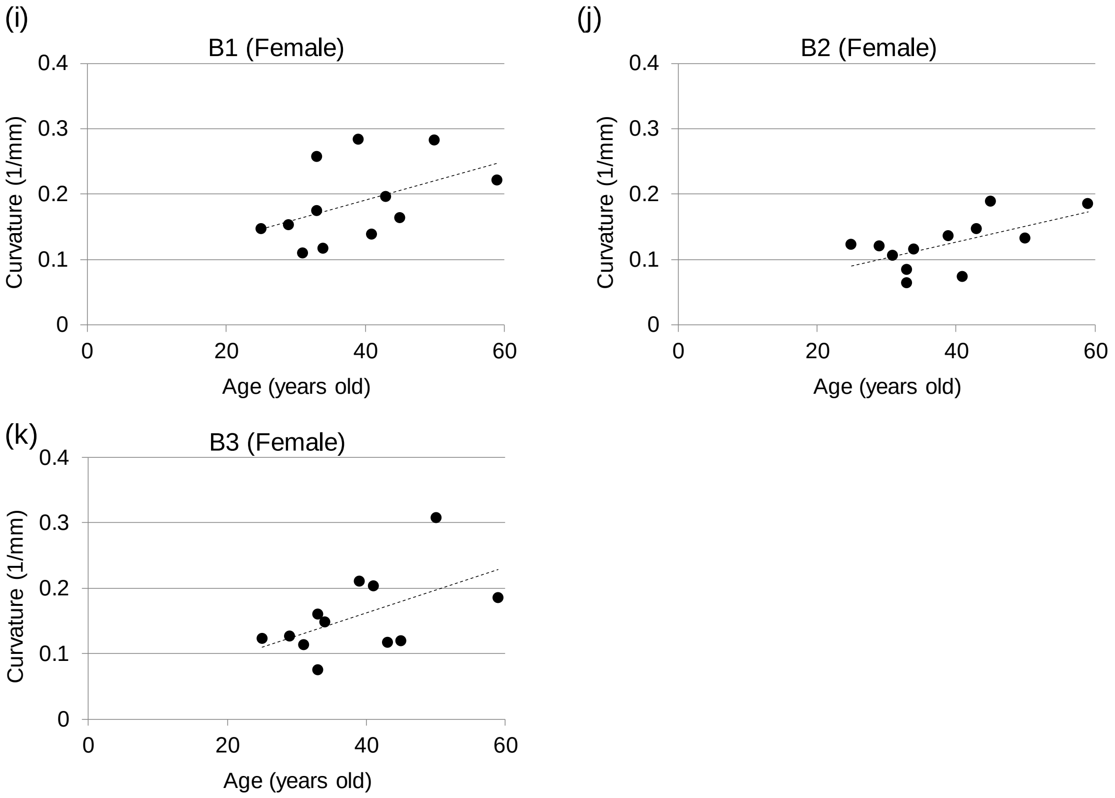
(i)
B1 (Female)
(j)
B2 (Female)
(k)
B3 (Female)
Curvature (1/mm)
Age (years old)
0
0.1
0.2
0.3
0.4
0
20
40
60

Fig. S6

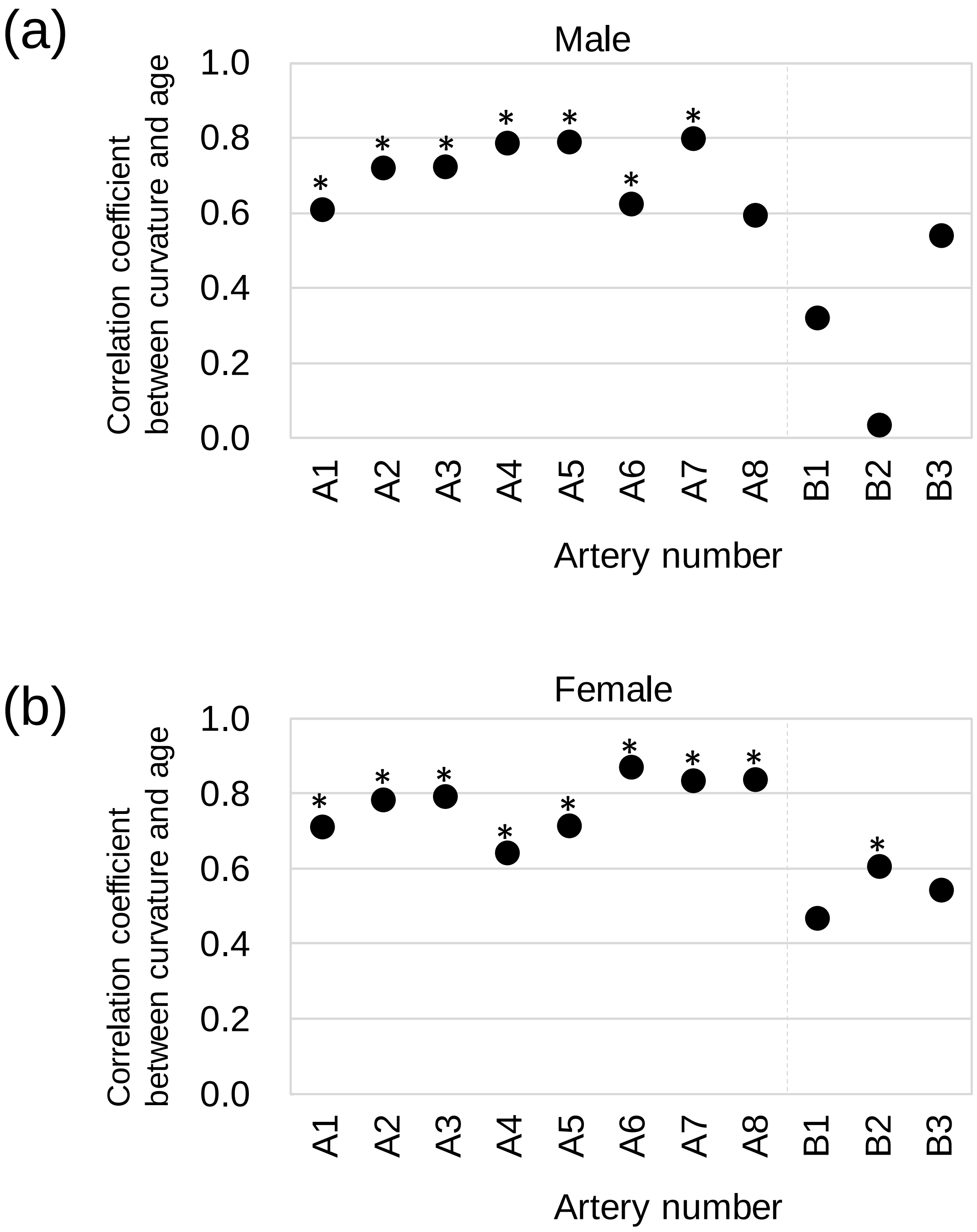
(a)
Male
Correlation coefficient between curvature and age
1.0
0.8
0.6
0.4
0.2
0.0
A1 A2 A3 A4 A5 A6 A7 A8 B1 B2 B3
Artery number
(b)
Female
Correlation coefficient between curvature and age
1.0
0.8
0.6
0.4
0.2
0.0
A1 A2 A3 A4 A5 A6 A7 A8 B1 B2 B3
Artery number

Fig. S7

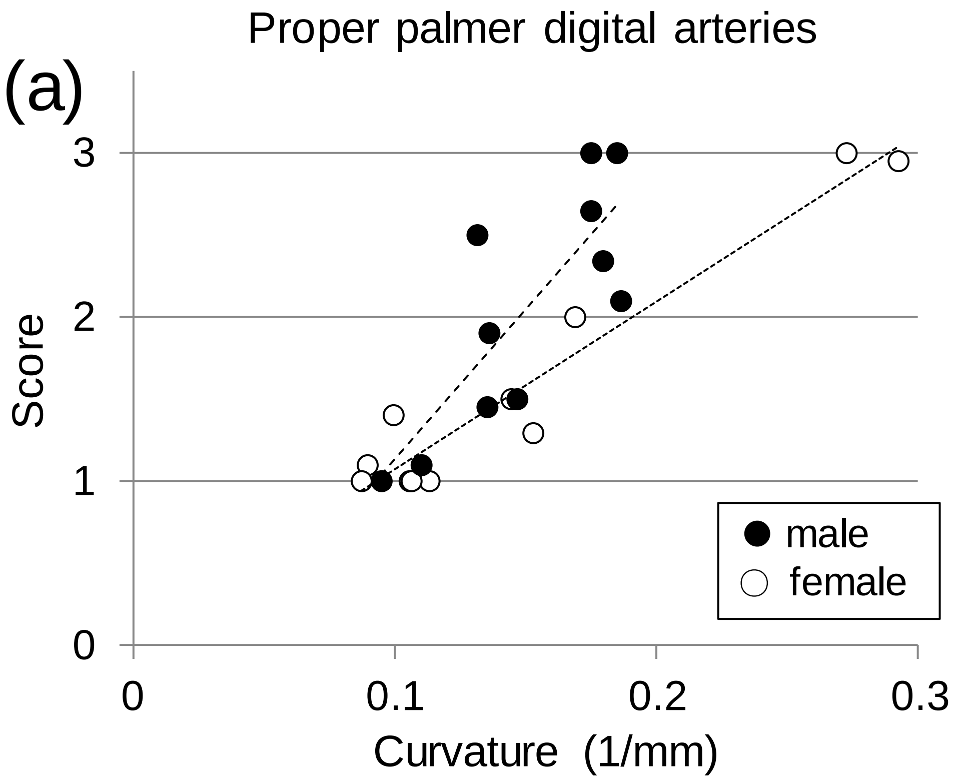
Proper palmer digital arteries
(a)
Score
3
2
1
0
male
female
0
0.1
0.2
0.3
Curvature (1/mm)

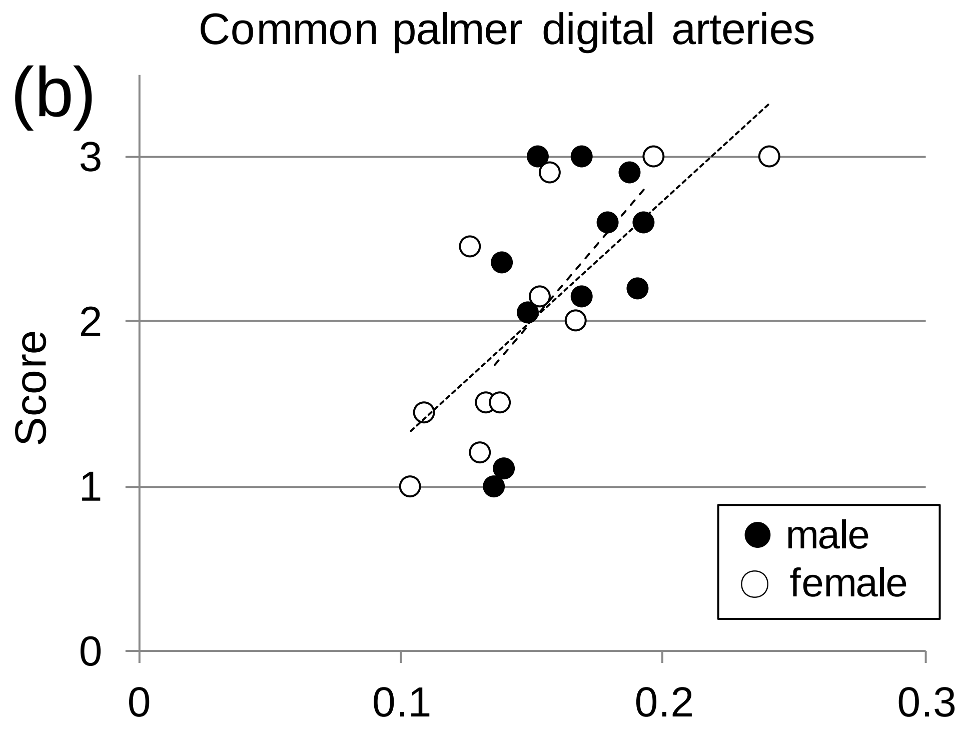
Common palmer digital arteries
(b)
Score
3
2
1
0
male
female
0
0.1
0.2
0.3

Fig. S8

Proper palmar digital arteries

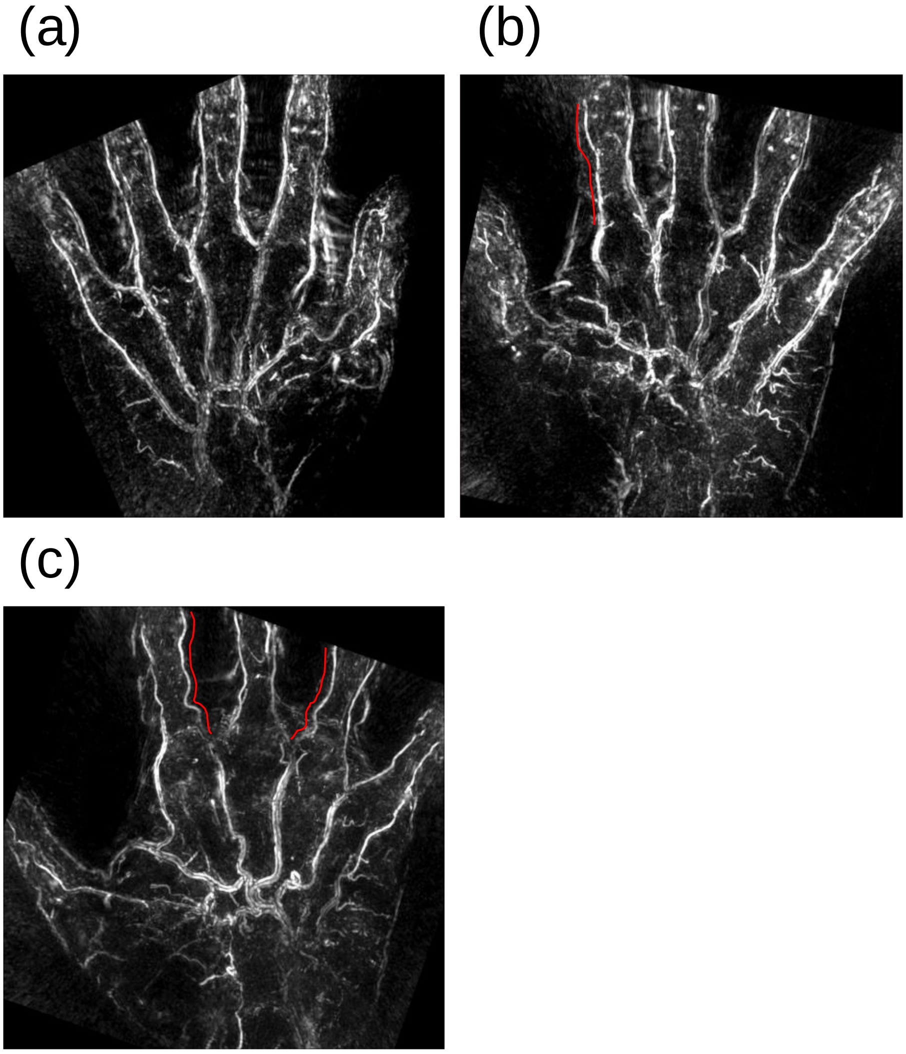

Fig. S9

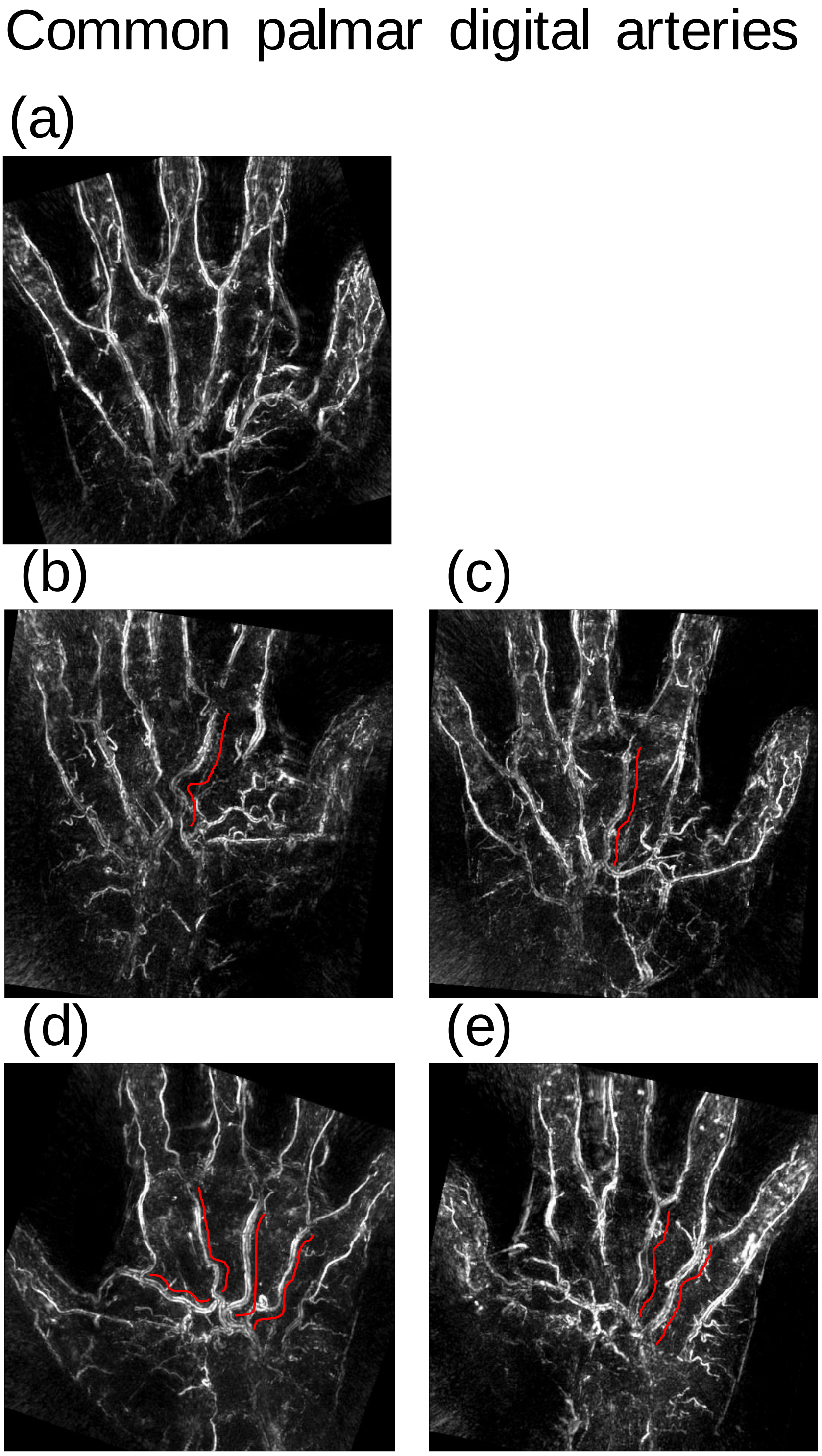

Supplement: Supplementary file 1 — Supplementary materials [file 41598_2018_19161_MOESM1_ESM.pdf]
